# Coomassie brilliant blue G 250 modified carbon paste electrode sensor for the voltammetric detection of dihydroxybenzene isomers

**DOI:** 10.1038/s41598-021-95347-2

**Published:** 2021-08-05

**Authors:** K. Chetankumar, B. E. Kumara Swamy, S. C. Sharma, H. Adarsha

**Affiliations:** 1 Department of P.G. Studies and Research in Industrial Chemistry, Kuvempu University, Jnana Sahyadri, Shivamogga (D), Shankaraghatta, Karnataka 577451 India; 2 National Assessment and Accreditation Council (Work Carried Out As Honorary Professor), Jain University, Bangalore, Karnataka 560 069 India; 3 Distinguished Professor in the School of Energy Science and Engineering, Indian Institute of Technology Guwahati, Guwahati, 781039 India; 4 Department of Mechanical Engineering, Faculty of Engineering and Technology, Jain Global Campus, Bengaluru, 562 112 India

**Keywords:** Biochemistry, Environmental sciences, Chemistry

## Abstract

In this present study, coomassie brilliant blue G-250 (CBBG) modified electrode was fabricated for the specific and simultaneous detection of three dihydroxybenzene isomers such as resorcinol (RS), catechol (CC) and hydroquinone (HQ). The fabrication of the modified electrode was carried out by electrochemical polymerization of CBBG on the surface of unmodified electrode. The surface structures of bare and fabricated electrode were studied by scanning electron microscope (SEM). The established electrode portrays the very fine interface with these isomers and displayed the sufficient sensitivity and selectivity. The specific parameters of pH solution, scan rate and varying the concentration of analytes were optimized at the modified electrode. The sensor process was originated to be adsorption-controlled activity and the low limit of detection (LOD) for RS and CC was attained at 0.24 and 0.21 µM respectively. In the simultaneous study, designed sensor clearly implies the three well separated anodic peaks for RS, HQ and CC nevertheless in unmodified electrode it failed. Also, the constructed electrode was applied for the real sample analysis in tap water and obtained results are agreeable and it consistent in-between 92.80–99.48%.

## Introduction

The three dihydroxybenzene isomers like resorcinol (RS), hydroquinone (HQ) and catechol (CC) are most vital intermediates of pharmacological industry and expansively utilized in preservatives, dye, antioxidants and stabilizer so on^1^. In US and European Union, they looked as environmental pollutants because they are more toxic and poor degradability in the ecological system^2–6^. For the purpose of community health and environment conservation it is essential to be scrutinized. Therefore, the quantitative examination of CC, RS and HQ was great importance. CC and HQ usually interfere with each other during their identification because of their similar structures and properties^7–10^. Therefore, analytical method was necessary for the simultaneous examination of CC, RS and HQ^11–16^. Several methods are reported for the tracing of CC, RS and HQ quantitatively, including voltammetry, calorimetry, gas chromatography-mass spectrum^17–21^ etc. In that electroanalytical technique, owing as a commanding technique because of its speedy reaction, supplementary sensitivity and selectivity, simple apparatus, and low cost^22–24^.

In this current study, Coomassie Brilliant Blue G-250 (CBBG) was used in the modification of CPE. CBBG was one of the utmost collective forms of coomassie dye. It is differing from Brilliant Blue R-250 and slightly greenish tint to its blue colour. It having some features like facile detection, great sensitivity, reversible staining, distinction between bound and unbound dye. The modification of CPE surface was carried out by electrochemical polymerization method using CV system and it is applied to the identification of RS and CC in the presence of HQ.

## Experimental section

### Equipment and chemicals

Voltammetric experiments are examined by CV and DPV techniques using a traditional three-electrode cell of CH Instrument-660 electrochemical workstation (CHI-660c). Bare Carbon Paste Electrode (BCPE) and poly(CBBG)/MCPE were utilized as working electrodes, saturated calomel electrode (SCE) as a reference electrode and a platinum wire as a counter electrode.

CC, RS and HQ gotten from Sigma Aldrich and standard solutions (25 × 10^–4^ M) was make ready in distilled water. CBBG, sodium dihydrogen phosphate, disodium hydrogen phosphate was procured from Merck chemicals and all aqueous solution (0.2 M) were prepared in distilled water. Pure graphite powder of 50 μM particle size from Merck and high viscous paraffin oil from fluka were used for the preparation of carbon paste. All the chemicals are used in this experimentation is with analytical grade and employed as received.

## Results and discussion

### Construction of bare and poly(CBBG)/MCPE

The BCPE was made-up by hand mixing of 30:70 (w/w) silicone oil and graphite powder for about 35 min and get homogeneous mixture. The gotten mixture was then filled with homemade Teflon cavity having 3 mm interior diameter and copper wire was used for the electrical contact.

**Figure 1:**
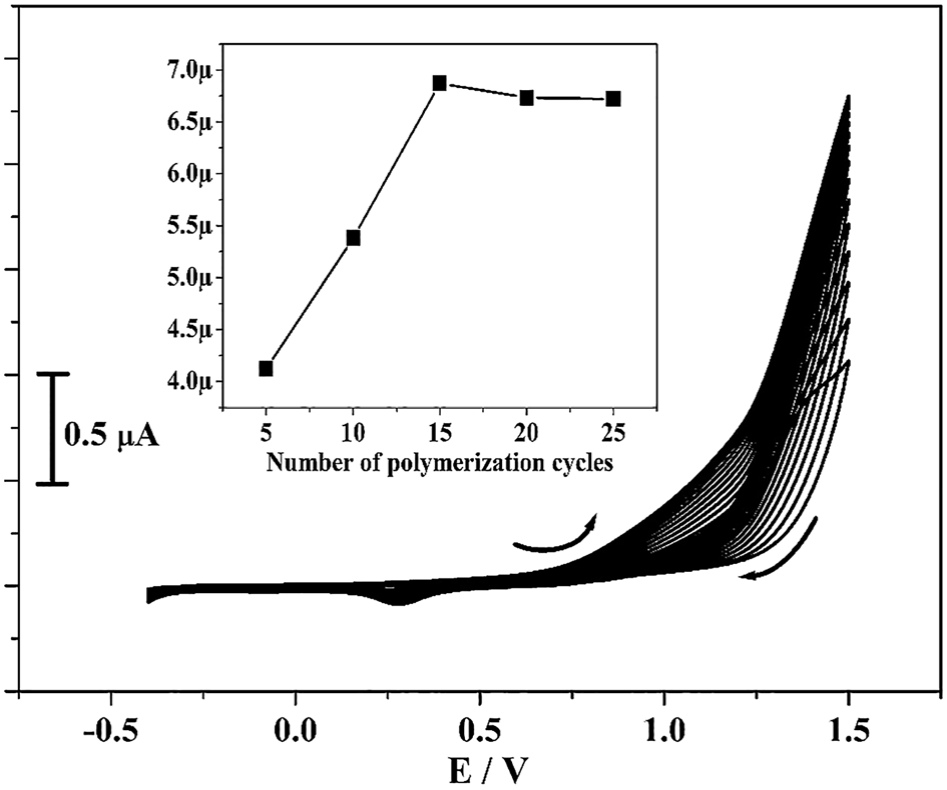
Electropolymerization of 2.0 mM coomassie brilliant blue G-250 on surface of BCPE in the existence of 0.1 M NaOH (supportive electrolyte) with scan rate 0.05 Vs^−1^ for 15 multiple cycles. Inset figure is plot of Ipa versus a number of various electropolymerization cycles.

**Scheme 1:**
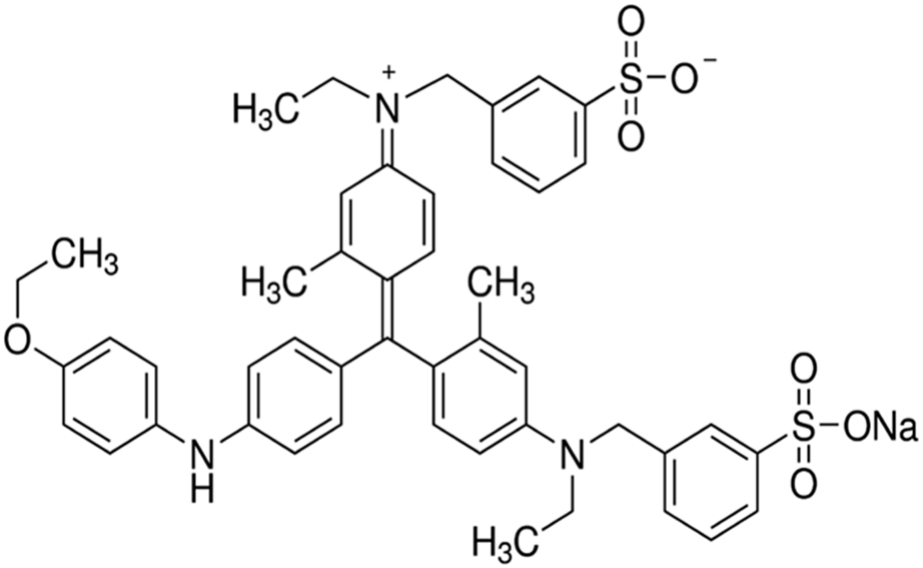
Structure of coomassie brilliant blue G-250.

Electrochemical polymerisation method was applied for the construction of modified electrode. CBBG (2.0 mM) was carried out on the surface of CPE using cyclic voltammetry in the presence of NaOH (0.1 M) and cyclic the potential in-between − 0.4 to 1.5 V with scan rate 0.05 Vs^−1^ for 15 polymerization cycles as shown in Fig. 1. As observed in figure, the peak current gradually enhanced with increasing the polymer cycles this confirms the growth of polymeric films on CPE^25,26^. The deposition of CBBG (Scheme 1) on CPE was made by changing the polymer cycles (5 to 25 cycles) and applied to identify the electrochemical performance towards CC (10 µM) in PBS (0.2 M) of pH 7.4. The fifteen polymerization cycles depict maximum enhancement in peak current signal revealed in inset Fig. 1. Thus, fifteen polymerization cycles were optimum for the modification of the electrode.

### Characteristics property and surface study of poly(CBBG)/MCPE

**Figure 2:**
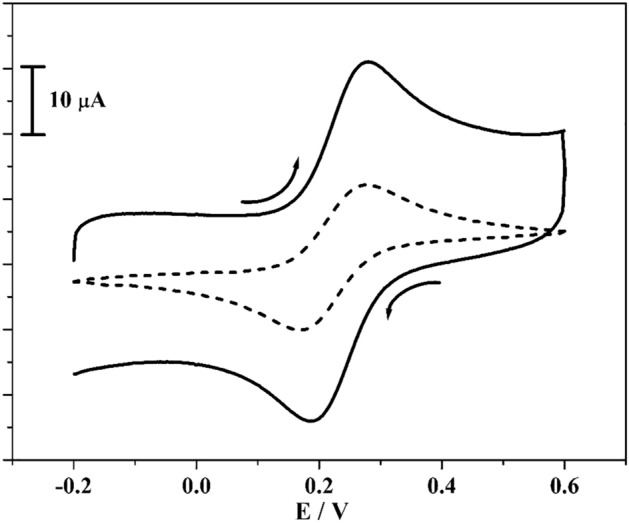
CVs curve recorded for 1 mM K_4_[Fe(CN)_6_] at unmodified (scattered streak) and poly (CBBG)/MCPE (hard streak) with sweep rate of 0.05 Vs^−1^ using 1 M KCl (supportive electrolyte).

The characteristic property of poly(CBBG)/MCPE was tracked by K_4_[Fe(CN)_6_] (25 × 10^–3^ M) in 1 M KCl (supporting media). Figure 2 represents the acquired cyclic voltammograms (CVs) for BCPE (scattered streak) and solid streak for the poly(CBBG)/MCPE with scan rate 50 mV/s. At BCPE, it indicates slight peak current and in the similar situation poly(CBBG)/MCPE exhibits superior growth in peak current than BCPE. Therefore, this enhancement in current gave great electroactive superficial area and this was determined using Randles-Sevick’s Eq. (1)^27^. Compared to unmodified CPE (0.027 cm^2^) the fashioned poly(CBBG)/MCPE (0.053 cm^2^) are accomplish more superficial surface area. The approximate adhered modifier thickness or surface average concentration on CPE was calculated using Eq. (2)^28,29^ and got at 0.183 × 10^−10^ M/cm^2^.

**Figure 3:**
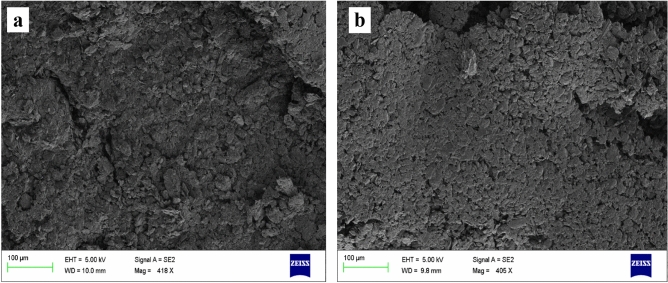
SEM pictures for BCPE (**a**) and poly (CBBG)/MCPE (**b**).

The surface changes of before and after modifying of CPE was characterized by SEM analysis using ZEISS Ultra-55. Figure 3a,b exposes the attained SEM pictures for BCPE and poly(CBBG)/MCPE. At BCPE, the layers shown as rough and wrinkle surface. After modification of CBBG on CPE it obvious changes was occurred and it looks like smooth and flat surface^30,31^.

1 $$Ip=2.69\times {10}^{5} {n}^{3/2} A {D}^{1/2}Co {\nu }^{1/2}$$

2 $$Ip={n}^{2}{F}^{2}A\Gamma \nu /4RT$$

where *C*_*0*_ is the concentration of the electroactive molecules (mol/cm^3^), *A* is the area of working electrode (cm^2^), *D* is the diffusion coefficient (cm^2^ s^− 1^), *n* is the transformed electrons, *ν* is the scan rate (V/s), *Ip* is the peak current, *Γ* (M/cm^2^) is the surface average concentration and R, F, T are the physical constants.

### Impact of pH on CC and RS at poly(CBBG)/MCPE

**Figure 4:**
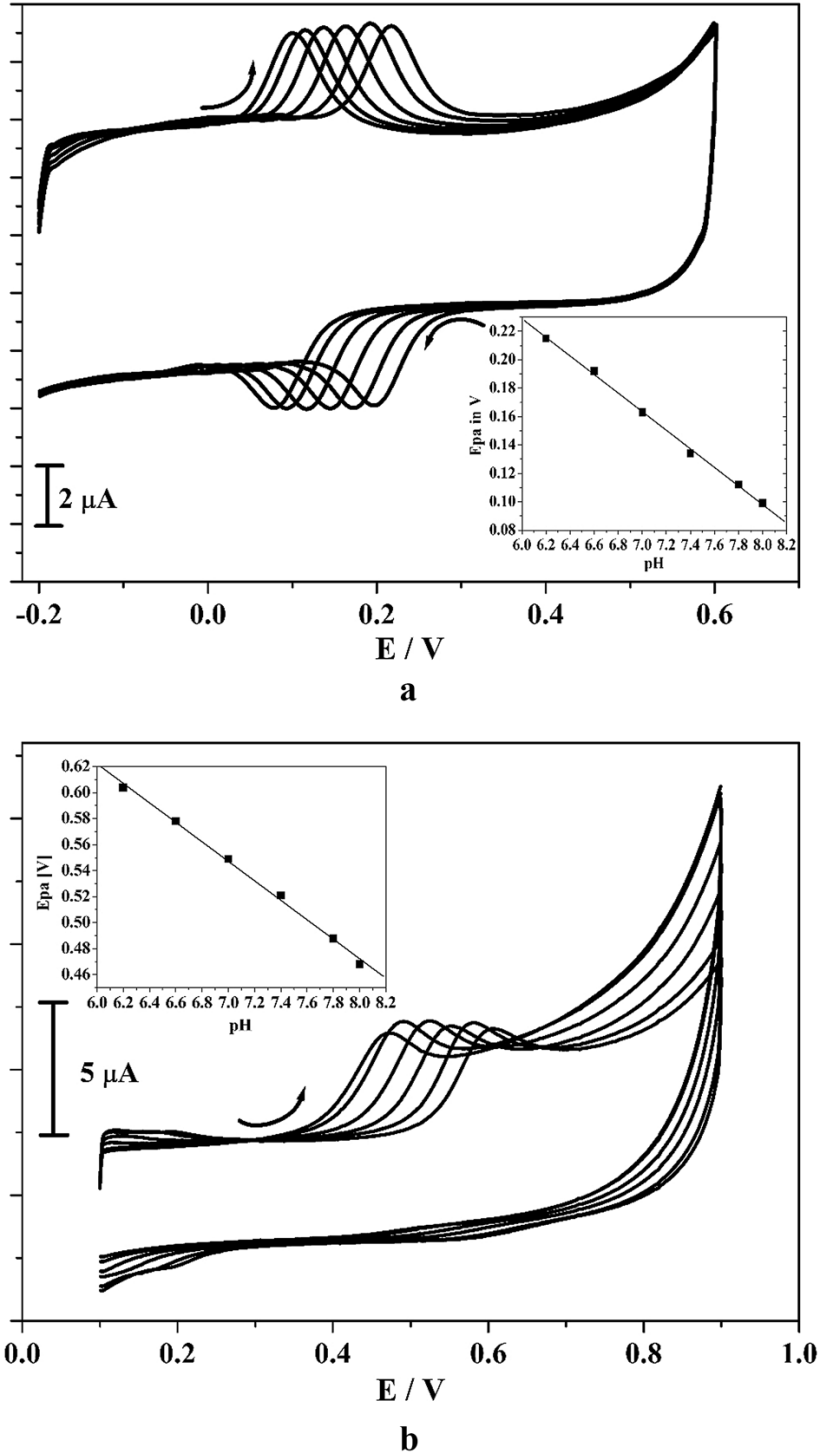
(**a**) CVs curve for 10 µM CC at poly (CBBG)/MCPE in the occurrence of altered pH and insert figure is plot of Epa versus pH. (**b**) CVs for 10 µM RS at poly (CBBG)/MCPE in presence of varied pH and inset figure is graph of Epa against pH.

The selection of supporting electrolyte takes principal role in an electrochemical activity. Figure 4a,b reveals the gotten CVs for CC and RS (10 μM) in occurrence of 0.2 M PBS of various pH (6.2, 6.6, 7.0, 7.4, 7.8, 8.0) with sweep rate of 0.05 V/s at poly(CBBG)MCPE. As observed in Fig. 4a,b, it clearly exposed that as the pH solution was varied then the peak potential (CC and RS) was moved towards the negative potential. This attained result was evidence for the directly involvement of proton in the electrochemical reaction^32,33^. The linear relationship between peak potential and varied pH solution for CC and RS was illustrated in inset Fig. 4a,b. The obtained regression equation is expressed for CC and RS as, Epa (V) = − 0.056 pH + 0.64 (R^2^ = 0.9991) and for RS, Epa (V) =  − 0.060 pH + 0.627 (R^2^ = 0.9993) respectively. The gotten slope value 56 and 60 mV/pH is very close to the Nernstian proposed value, therefore it clearly recommends the equivalent number of electron and proton was participated in the electrode response^34,35^. By considering the sensitivity, pH 7.4 solution was optimised for additional electrochemical investigations.

### Cyclic voltammetric performance of CC on poly(CBBG)/MCPE

**Figure 5:**
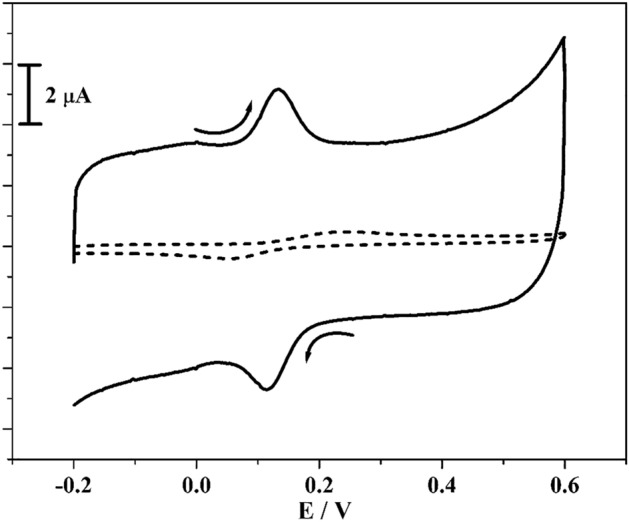
CVs curve 10 µM CC at unmodified CPE (scattered streak) and poly(CBBG)/MCPE (dashed streak) with scan rate 0.05 Vs^-1^ using 0.2 M PBS of pH 7.4.

The identifying capability of established sensor towards the CC was examined by CV technique as more sensitive and accurate electroanalytical techniques. In Fig. 5 the scattered line and hard line depicts CVs for BCPE and poly(CBBG)/MCPE of CC (10 μM) in the existence of PBS (0.2 M) with scan rate 0.05 Vs^−1^. The BCPE provides CVs with poor and wide peaks and in the same state constructed electrode gave very fine sensitivity with sharp peaks. Compared to BCPE the fabricated electrode implies supreme enrichment in peak current. The peak potential variance (ΔEp) was acquired at 0.148 V (BCPE) and 0.019 V (poly(CBBG)/MCPE) respectively.

**Scheme 2:**
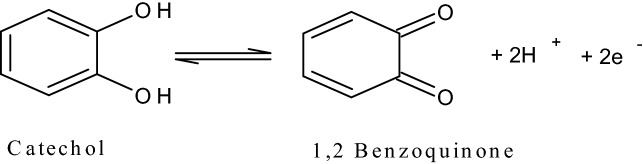
Redox reaction of CC.

The transfer of electron was easier in constructed electrode than BCPE, because where ΔEp value is lower and then electron transfer rate will be greater. Therefore, the poly(CBBG)/MCPE act as moral and prominent sensor for the investigation of CC. The oxidation and reduction mechanism of CC was portrayed in Scheme 2.

### Effect of scan rate and concentration variation on peak current of CC at poly(CBBG)/MCPE

**Figure 6:**
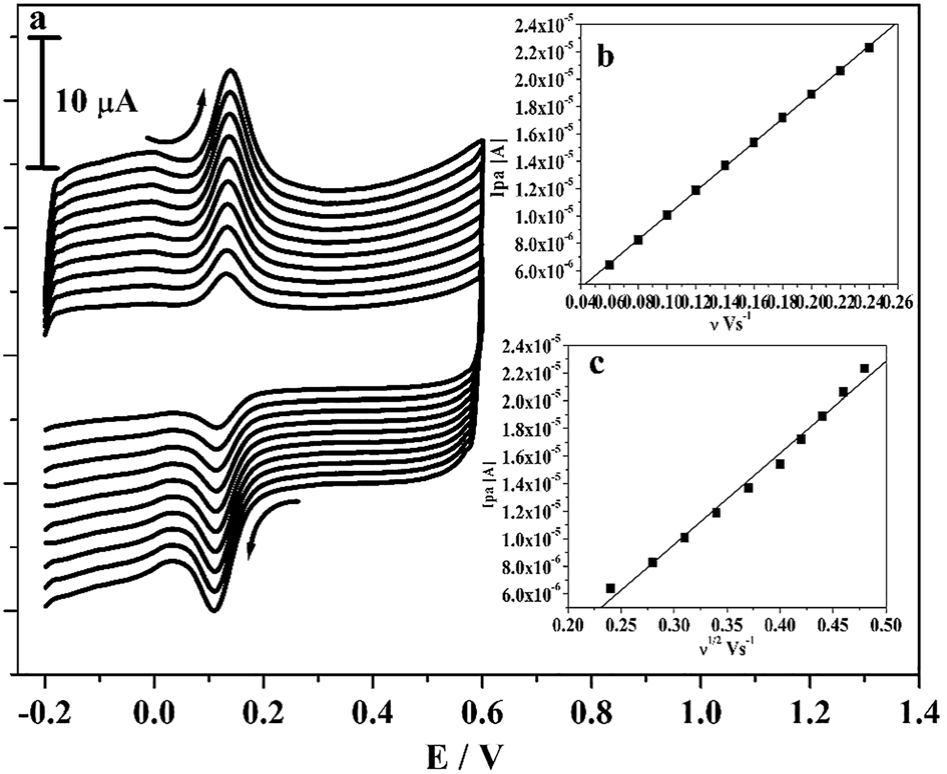
(**a**) CVs curve for 10 µM CC at poly(CBBG)/MCPE with altered sweep rates (0.06–0.24 Vs^−1^) in 0.2 M PBS of pH 7.4. (**b**) Plot of Ipa versus scan rate. (**c**) Plot of Ipa versus square root of scan rate.

The kinetics of poly(CBBG)/MCPE was examined by changing the scan rate. Figure 6a illustrated the obtained CVs for 10 μM CC in the existence of 0.2 M PBS (supporting electrolyte) with changed scan rates. As perceived in figure, the peak current of CC was subsequently enhanced with as raised in the scan rate (0.06 to 0.24 V/s) and tiny moved of their peak potential to positive and negative potential. The linear correlation between anodic peak current (Ipa) versus scan rate (ν) and Ipa versus square root of the scan rate was drawn in Fig. 6b,c. The gotten graph gave very fine linearity with correlation coefficient value (R^2^) was originated to 0.999 and 0.998 respectively. Therefore, by noticing the above achieved outcome the kinetic characteristic of poly(CBBG)/MCPE was originated at surface controlled response^34,36^.

**Figure 7:**
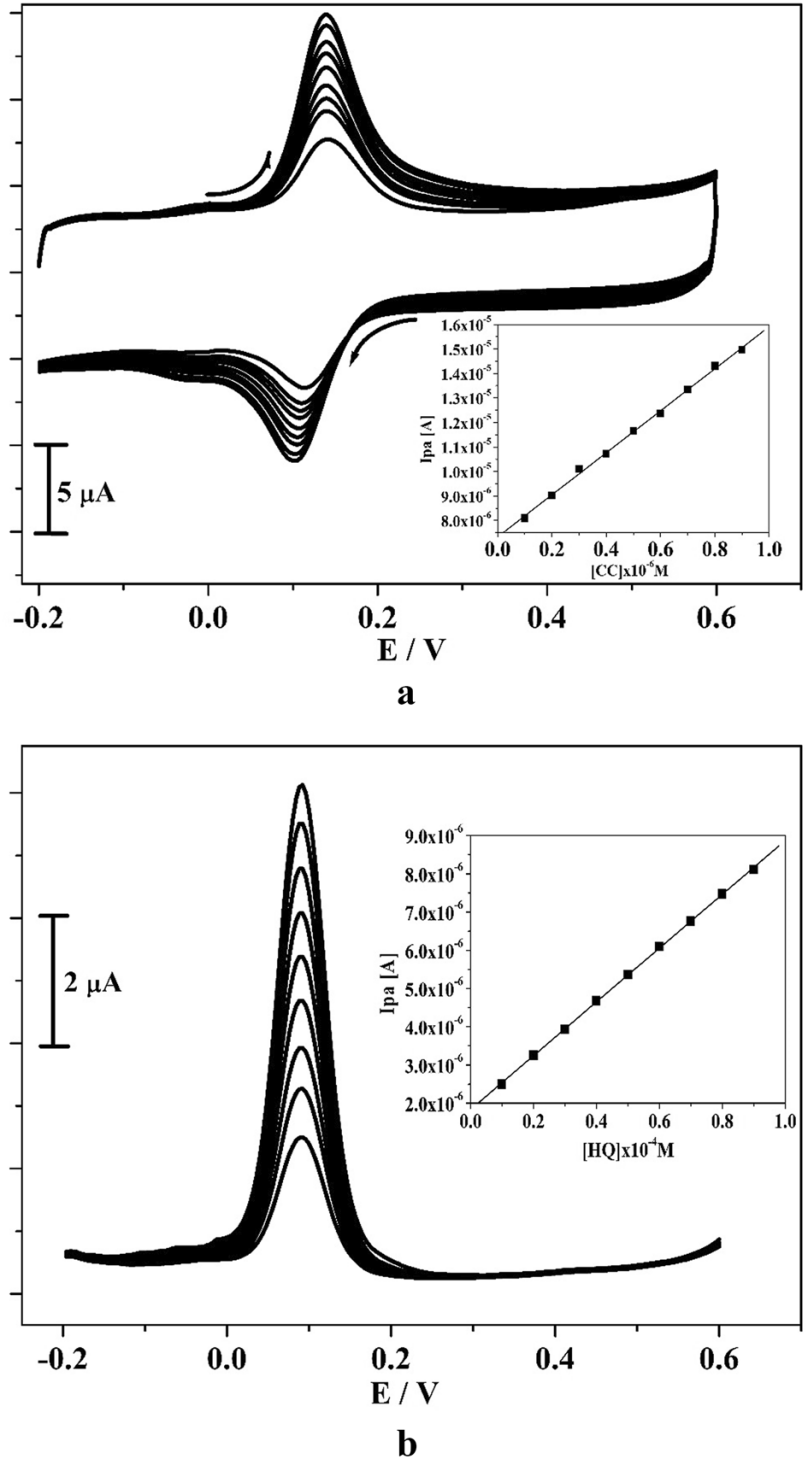
(**a**) CVs curve for CC at poly(CBBG)/MCPE with various concentration (10–90 µM) in presence 0.2 M PBS (pH 7.4) with scan rate of 50 mVs^-1^ and inset figure is plot of Ipa versus CC concentration. (**b**) DPVs for altered concentration of CC (10–90 µM) at poly(CBBG)/MCPE and inset figure is plot of Ipa against CC concentration.

**Figure 8:**
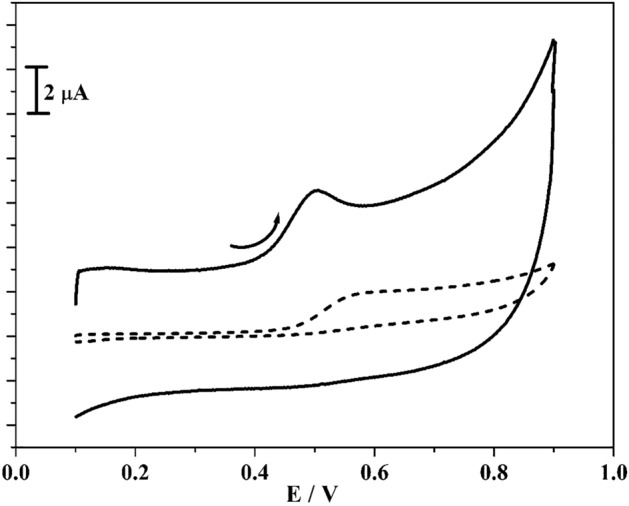
CVs curve for 10 µM RS in 0.2 M PBS (pH 7.4) at BCPE (scattered streak) and poly(CBBG)/MCPE (hard streak) with scan rate of 0.05 Vs^−1^.

The detection limit of amended electrode was assessed by operating CV and DPV performance. Figures 7a and 8b denotes the gained CVs and DPVs for different concentration of CC (10–90 μM) in the occurrence of 0.2 M PBS of pH 7.4 with scan rate 0.05 V/s. These figures clearly depict that the peak current was enhanced significantly when the concentration of analyte was increases. Inset Fig. 7a,b implies the correlation of Ipa and analyte concentration and it gave sufficient linearity with R^2^ value of 0.999. By applying the standard deviation (S) and slope value (M) of the peak current (acquired from inset Fig. 7a,b) evaluated the LOD and LOQ by utilizing Eqs. (3 and 4)^6,37^ for CC and gotten to be 0.21 and 0.69 μM individually.

3 $${\text{LOD}} = {\text{3S}}/{\text{M}}$$

4 $${\text{LOQ}} = {1}0{\text{S}}/{\text{M}}$$

### Electrochemical response, effect of scan rate and concentration variation of RS on poly(CBBG)/MCPE

**Scheme 3:**
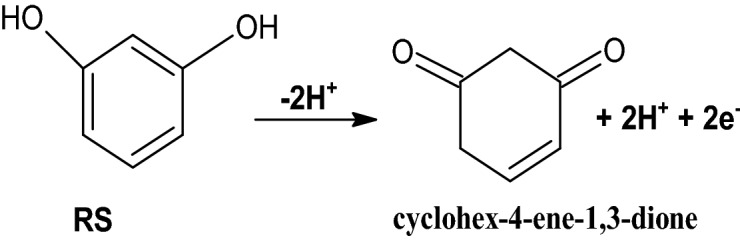
Redox response of RS.

The voltammetric response of RS was tracked by CV technique and Fig. 8 illustrates the acquired CVs curve for RS (10 μM) in the occurrence of 0.2 M PBS (pH 7.4) with the scan rate 0.05 Vs^−1^ at poly(CBBG)/MCPE. At BCPE (scattered streak) it depicts slight peak current with oxidation potential of 0.56 V is resembles to the oxidation of RS. Likewise, in the unchanged condition the established poly(CBBG)/MCPE (hard streak) gave superior enlargement in oxidation peak current for RS than unmodified CPE with tiny moved their oxidation potential (0.50 V). Scheme 3 signifies the oxidation response of RS.

**Figure 9:**
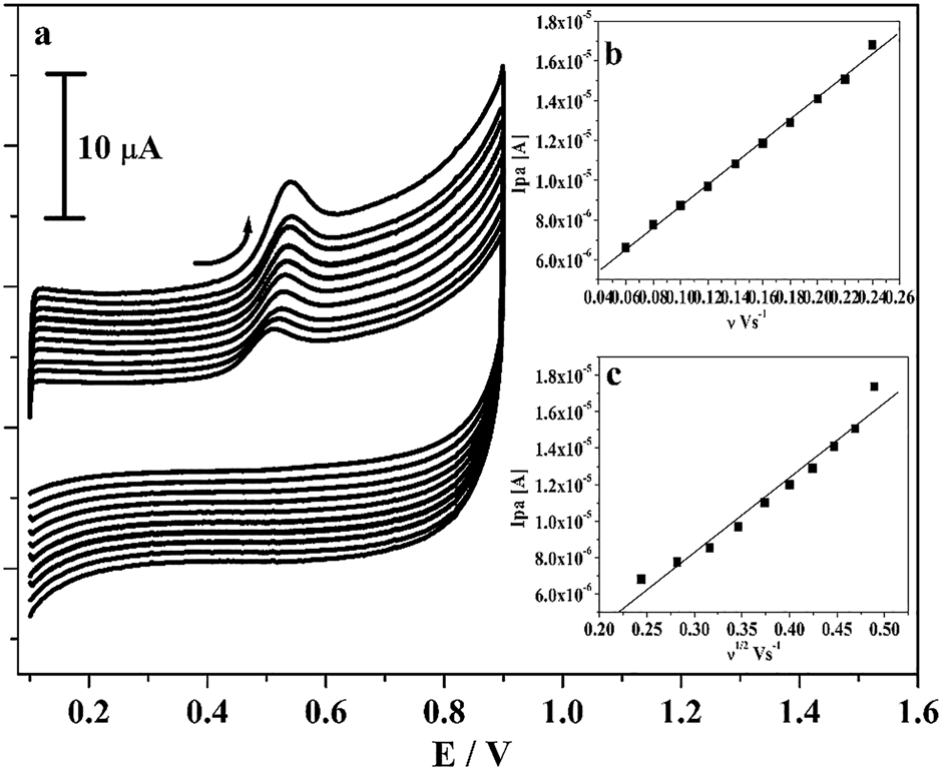
(**a**) CVs curve for 10 µM RS in 0.2 M PBS (pH 7.4) at poly(CBBG)/MCPE with varied scan rates (0.06–0.24 Vs^−1^). (**b**) Graph of Ipa against scan rate. (**c**) Graph of Ipa against square root of scan rate.

The variant of scan rate study gave the needed evidence for the process of electrode. The Fig. 9a displayed the received CVs for 10 μM RS in the existence of 0.2 M PBS (pH 7.4) with various scan rate (0.06 to 0.24 V/s). By observing the Fig. 9a, as the scan rates elevated the oxidation peak current was enhanced subsequently by tiny slide in their oxidation peak potential to positive potential. The interrelation between scan rate and oxidation peak current and square root of scan rate versus oxidation peak current is plotted in Fig. 9b,c respectively. The gotten graph gave fine linearity with R^2^ was gotten at 0.998 and 0.995 respectively and the process of electrode was originated by surface-controlled phenomena.

**Figure 10:**
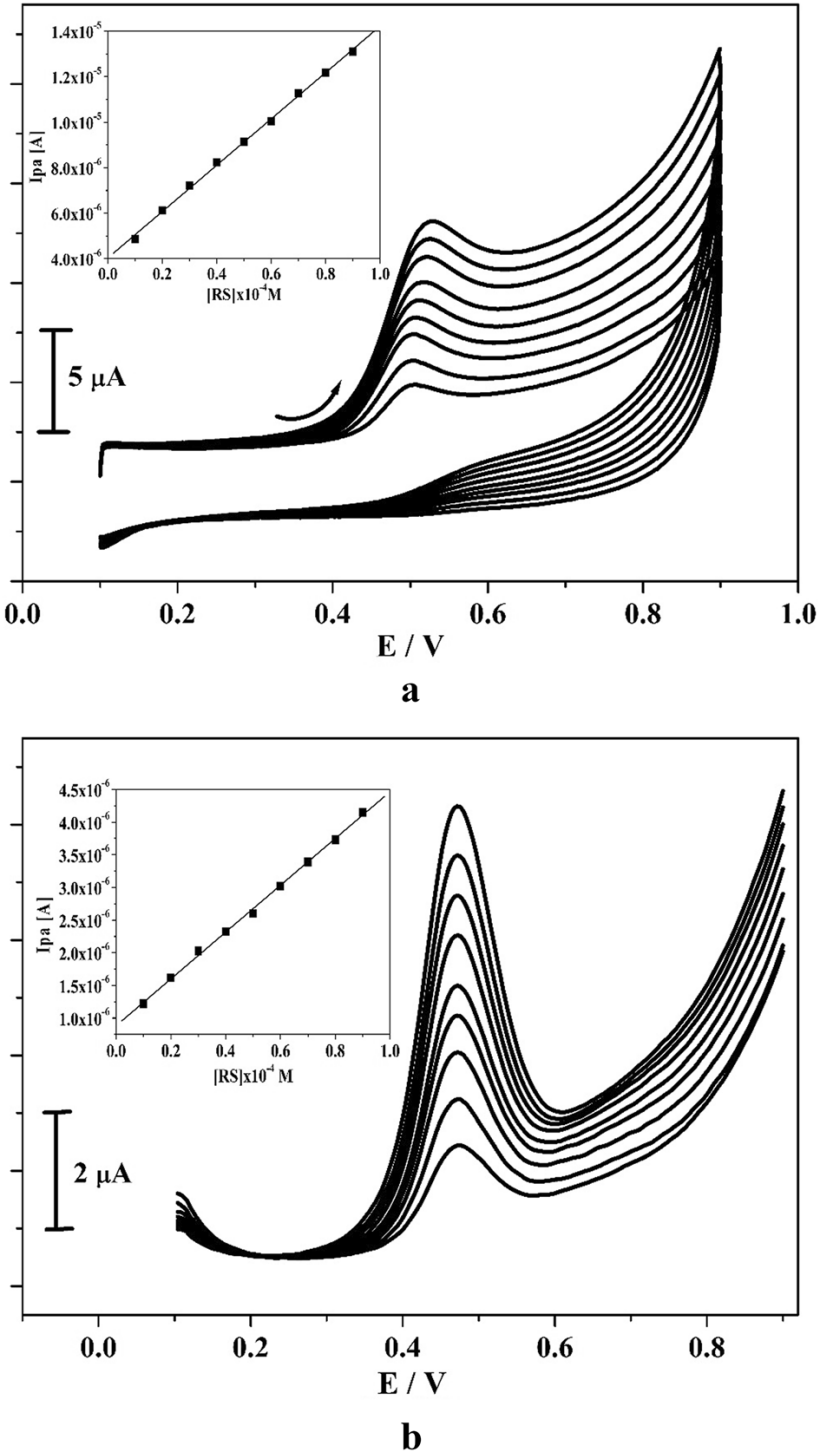
(**a**) CVs for various concentration of RS (10–90 µM) at poly(CBBG)/MCPE with using 0.2 M PBS (pH 7.4) with scan rate of 50 mVs^-1^ and inset figure is plot of Ipa versus CC concentration. (**b**) DPVs curve for changed concentration of RS (10–90 µM) at poly(CBBG)/MCPE and inset figure is plot of Ipa and concentration of RS.

LOD and LOQ was assessed by the Eqs. (3 and 4) by applying the CV and DPV technique. The gotten voltammograms for concentration variation of RS (10–90 μM) in the existence of 0.2 M PBS (pH 7.4) with scan rate 0.05 Vs^−1^ was demonstrated in Fig. 10a,b. As noticed in Fig. 10a,b, the oxidation peak current of RS was increases subsequently as the concentration raises and peak potential tiny moving to positive direction. The inset Fig. 10a,b depicts the correlation between concentration of RS and oxidation peak current and it offered very satisfactory linearity with R^2^ value is 0.997. The gotten LOD and LOQ for RS is 0.24 and 0.79 μM separately. The poly(CBBG) MCPE gave less detection limit for CC and RS compared to other fabricated electrode shown in Table 1.

**Table 1:** Comparison of LOD at poly(CBBG)/MCPE with other reported modified electrodes for the determination CC, RS and HQ.

Working electrode	Limit of detection (µM)		References
	**CC**	**RS**	
Graphene-CS/GCE	0.75	0.75	^42^
Au-PdNF/rGO/GCE	0.8	0.7	^43^
CoPC/PGE	0.34	0.72	^44^
poly(hydroxynaphthol blue)/MWCNT/GCE	0.24	0.26	^45^
Poly(3-Thiophenemalonic Acid)/MGCE	3.91	15.6	^46^
Ammoniated-PBS/GCE	0.23	0.47	^47^
Poly(vanillin)/MCPE	0.95	–	^48^
**Poly(CBBG) MCPE**	**0.21**	**0.24**	**Present work**

### Simultaneous voltammetric detection of CC and RS in the presence of HQ at poly(CBBG)/MCPE

**Figure 11:**
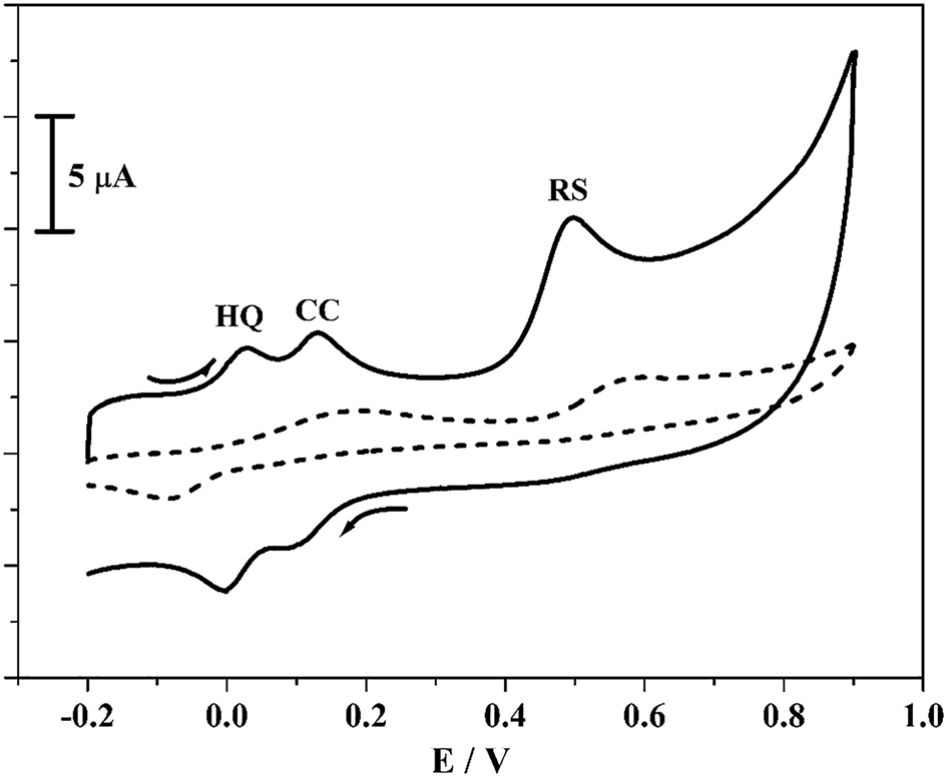
CVs curve for simulataneous examination of CC, RS and HQ (10 µM) in 0.2 M PBS of pH 7.4 at BCPE (scattered streak) and poly(CBBG)/MCPE (hard streak) with scan rate 0.05 Vs^−1^.

The simultaneous detection of CC, RS and HQ was very problematic in mixed solution at BCPE. As in the blending solution they are amalgamate each other owing to their closely similar oxidation potential^38^. Therefore, to validate the potentiality of modified electrode for the simultaneous investigations of CC and RS in the presence of HQ. Figure 11 reveals the gotten CVs for CC, RS and HQ (10 μM) in the existence of supportive electrolyte (0.2 M PBS of pH 7.4) with scan rate 0.05 Vs^−1^. The BCPE (scattered streak) was unsuccessful to depicts distinguished the three peaks but it gave two oxidation peaks situated at 0.15 and 0.56 V correspondingly. Moreover, the established poly(CBBG)/MCPE (dashed line) clearly demonstrated the three separated oxidation peaks for CC, RS and HQ (0.13, 0.49 and 0.02 V) individually with exceptional improvement in peak current than BCPE. Therefore, the fabricated poly(CBBG)/MCPE was remarkable potentiality towards the simultaneous detection for CC and RS in the existence of HQ.

### Selectivity, stability and real sample exploration of CC, RS and HQ at poly(CBBG)/MCPE

**Figure 12:**
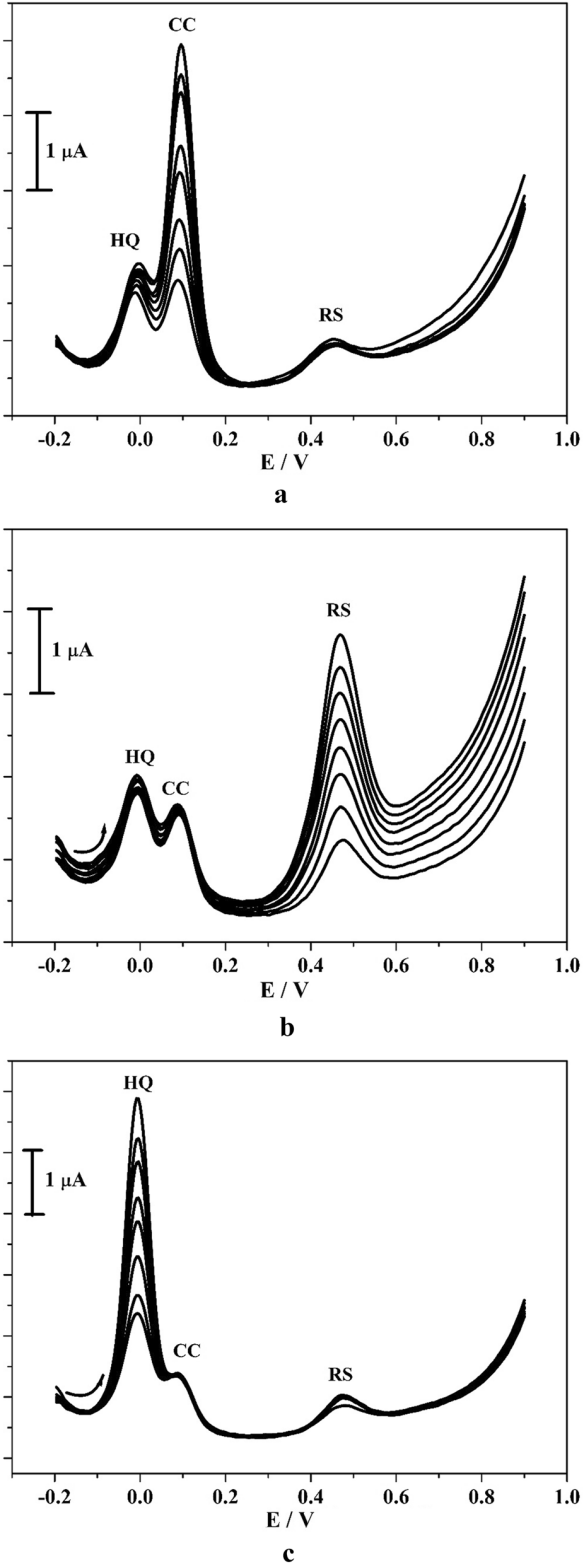
(**a**) DPVs curve for CC with various concentration (100–800 µM) in presence of HQ and RS (10 µM). (**b**) DPVs curve for RS with various concentration (100–800 µM) in presence of CC and HQ (100 µM). (**c**) DPVs curve for altered concentartion of HQ (100–800 µM) in presence of RS and CC (100 µM) in 0.2 M PBS (pH 7.4) at poly(CBBG)/MCPE with scan rate 50 mVs^-1^.

The efficiency and selectivity detection of CC, RS and HQ was examined by utilizing the DPV system. Figure 12a represents the tracing of CC by reserved the concentration of RS and HQ (50 μM) was unchanged. As perceived in the figure, the peak current was enhanced by increasing the concentration of CC in the series 100–400 μM. Similarly, for RS the amount of analyte was varied in the series 100 to 400 μM by kept the unchanged solution of HQ and CC (50 μM) signified in Fig. 12b. Likewise, the amount of concentration for HQ (50–400 μM) was varied and the amount of RS and CC (50 μM) was unchanged. By seeing the overhead outcome, as the amount of concentration was enlarged, the peak current also enhanced gradually, nevertheless there was nothing variation in peak potential and current of reserved mixtures. This consequence specifies that the reserved analytes are no restrict in detection of mixtures. Thus, poly(CBBG)/MCPE consume admirable selectivity and they are traced individually in blended solution.

**Figure 13:**
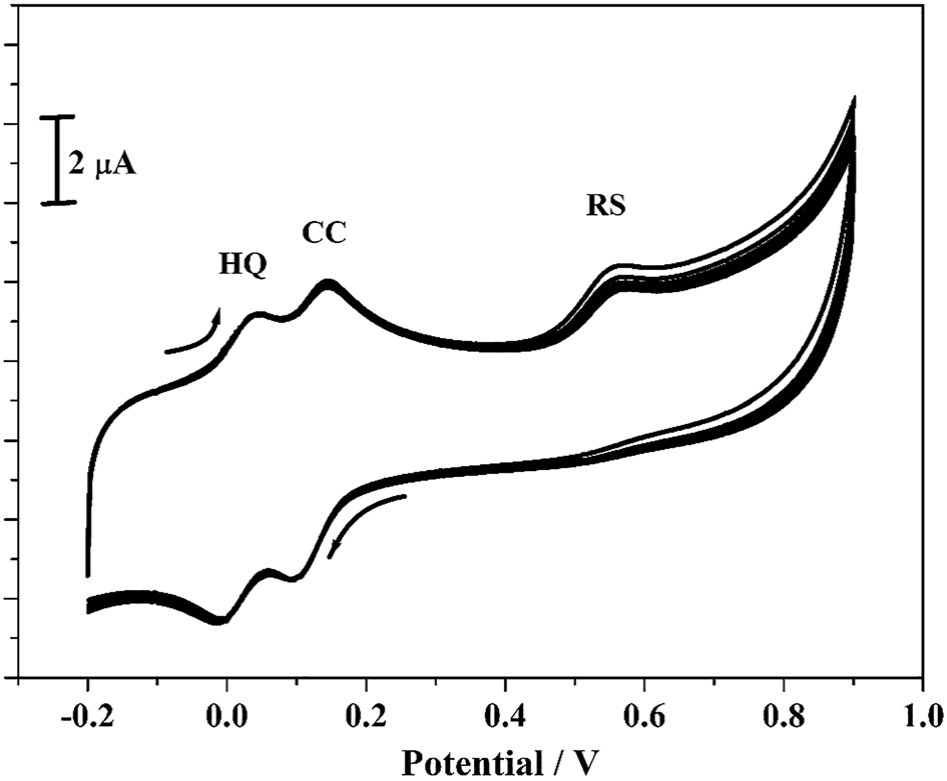
CVs of CC, RS and HQ (10 µM) in the presence of 0.2 M PBS (pH 7.4) with scan rate 0.05 V/s at poly(CBBG) MCPE for 10 cycles.

The steadiness of the poly(CBBG)/MCPE was estimated in RS, CC and HQ (10 µM) in 0.2 M PBS (pH 7.4) with the sweep rate 0.05 Vs^−1^ in ten consecutive cycles applying CV technique. As in the Fig. 13, the redox peak current remains steady and after accomplishment of 10 cycle the slight reductions in their oxidation current were 4.9%. The degradation percentage was estimated by equation % degradation = Ip_n_/Ip_1_^39^, where Ip_1_ and Ip_n_ are the 1^st^ and n^th^ cycle for Ipa separately. The regained steadiness of the poly(CBBG)/MCPE was got at 95.10% and this approves the fashioned electrode consumed adequate stability.

Finally, the recommended poly(CBBG)/MCPE was examined for the real sample applicability of CC, RS and HQ in a tap water utilizing standard addition method^40,41^. This advised the fabricated electrode portrays satisfactory retrieval for each sample adding and acquired outcomes are registered in Table 2. Therefore, this outcome authorized the accurateness of established electrode for the revealing CC, RS and HQ in tap water.

**Table 2:** Recoveries of CC, RS and HQ in a local tap water sample at poly(CBBG)/MCPE.

Sample	Compound	Added (µM)	Founded (µM)	Recovery (%)
**Tap water**	**CC**	15	14.20	94.66
		25	23.61	94.44
		35	34.61	98.88
	**RS**	15	14.64	97.60
		25	23.21	92.84
		35	33.97	97.05
	**HQ**	15	13.92	92.80
		25	24.87	99.48
		35	34.07	97.34

## Conclusion

Present work outlined, poly(CBBG)/MCPE was operated as the sensor for the detection of CC and RS in presence of HQ. The surface property of BCPE and poly(CBBG)/MCPE was scanned by SEM exploration. This suggested probe exposed the strong electro-catalytic movement, great sensitivity, steadiness and gave improved electron transfer reaction than BCPE with respect to the oxidation of CC and RS in the presence of HQ. The influence of pH, analyte concentration variation and scan rate were surveyed at fashioned electrode. The poly(CBBG)/MCPE implies surface controlled manner and it exhibited less detection limits than other testified electrode. The sensor has a wide dynamic range that comprises both linear ranges and simultaneous tracking of CC and RS in the presence of HQ was tracked via CV technique. The adopted method of established electrode contributed noble selectivity, stability and gave agreeable retrieval in the real sample scrutiny of analytes. Hence, the mentioned poly(CBBG)/MCPE was remarkable potentiality for the specific and simultaneous analysis. This fabricated electrode can also be utilized for the supplementary examination of other electroactive molecules also.

## References

[1] Chetankumar K, Swamy BEK, Sharma SC (2020). Electrochemical preparation of poly (direct yellow 11) modified pencil graphite electrode sensor for catechol and hydroquinone in presence of resorcinol: A voltammetric study. Microchem. J..

[2] Amare M, Begashaw M (2019). Fe(III) doped zeolite-graphite composite modified glassy carbon electrode for selective voltammetric determination of resorcinol in water samples. Microchem. J..

[3] Zhang M, Ye J, Fang P, Zhang Z, Wang C, Wu G (2019). Facile electrochemical preparation of NaOH nanorods on glassy carbon electrode for ultrasensitive and simultaneous sensing of hydroquinone, catechol and resorcinol. Electrochim. Acta.

[4] Sarvajith MS, Mruthyunjayachari CD, Harish MN, Kotresh K, F. (2002). Silver nanoparticles decorated phthalocyanine doped polyaniline for the simultaneous electrochemical detection of hydroquinone and catechol. J. Electroanal. Chem..

[5] Zhao H, Wang Y, Qian S, Fu XuC, Li W, Li X, Su D, Hu Z (2019). Graphite-doped polyimide films for sensitive non-enzymatic amperometric determination of catechol. Int. J. Electrochem. Sci..

[6] Ma Y, Cao Z, Wang Y, Xia Y, He C, Wang L, Bao S, Yin P, Wang L, Gao J, Wang H, Yin Z (2019). Simultaneous determination of catechol and hydroquinone using N, P co-doped carbon derived from ionic liquid. Int. J. Electrochem. Sci..

[7] Chetankumar K, Swamy BEK, Naik TSSK (2020). A reliable electrochemical sensor for detection of catechol and hydroquinone at MgO/GO modified carbon paste electrode. J. Mater. Sci. Mater. Electron..

[8] Chen H, Wu X, Lao C, Li Y, Yuan Q, Gan W (2019). MOF derived porous carbon modified rGO for simultaneous determination of hydroquinone and catechol. J. Electroanal. Chem..

[9] Peng Y, Tang ZR, Dong YP, Che G, Xin ZF (2018). Electrochemical detection of hydroquinone based on MoS2/reduced graphene oxide nanocomposites. J. Electroanal. Chem..

[10] Chetankumar K, Swamy BEK (2020). Electrochemically nitric acid pre-treated glassy carbon electrode sensor for catechol and hydroquinone: A voltammetric study. Sensors Int..

[11] El-Ads EH, Atta NF, Galal A, Eid NA (2018). electrochemical sensor based on nano-perovskite/ionic liquid crystal modified carbon paste electrode for effective determination of hydroquinone and catechol. Int. J. Electrochem. Sci..

[12] Feng X, Gao W, Zhou S, Shi H, Huang H, Song W (2013). Discrimination and simultaneous determination of hydroquinone and catechol by tunable polymerization of imidazolium-based ionic liquid on multi-walled carbon nanotube surfaces. Anal. Chim. Acta.

[13] Xiang Y, Li L, Liu H, Shi Z, Tan Y, Wu C, Liu Y, Wang J, Zhang S (2018). One-step synthesis of three-dimensional interconnected porous carbon and their modified electrode for simultaneous determination of hydroquinone and catechol. Sensors Actuators B: Chem..

[14] Tsai MS, Lu CJ, Su PG (2018). One-pot synthesis of AuNPs/RGO/WO3 nanocomposite for simultaneously sensing hydroquinone and catechol. Mater. Chem. Phys..

[15] Nazari M, Kashanian S, Moradipour P, Maleki N (2018). A novel fabrication of sensor using ZnO-Al2O3 ceramic nanofibers to simultaneously detect catechol and hydroquinone. J. Electroanal. Chem..

[16] Chen T, Xu J, Arsalan M, Sheng Q, Zheng J, Cao W, Yue T (2019). Controlled synthesis of Au@Pd core-shell nanocomposites and their application for electrochemical sensing of hydroquinone. Talanta.

[17] Nagaraja P, Vasantha RA, Sunitha KR (2001). A sensitive and selective spectrophotometric estimation of catechol derivatives in pharmaceutical preparations. Talanta.

[18] Si W, Lei W, Zhang Y, Xia M, Wang F, Hao Q (2012). Electrodeposition of graphene oxide doped poly(3,4-ethylenedioxythiophene) film and its electrochemical sensing of catechol and hydroquinone. Electrochim. Acta.

[19] Si W, Lei W, Hao Q, Zhang Y, Xia M (2014). Selective sensing of catechol and hydroquinone based on poly(3,4-ethylenedioxythiophene)/nitrogen-doped graphene composites. Sensors Actuators B: Chem..

[20] Yang P, Zhu Q, Chen Y, Wang F (2009). Simultaneous determination of hydroquinone and catechol using poly(p-aminobenzoic acid) modified glassy carbon electrode. J. Appl. Polym. Sci..

[21] Cogal S (2018). Electrochemical determination of dopamine using a poly (3, 4-ethylene-dioxythiophene)-reduced graphene oxide-modified glassy carbon electrode. Anal. Lett..

[22] Naik TSSK, Swamy BEK (2018). Pre-treated glassy carbon electrode sensor for catechol: A voltammetric study. J. Electroanal. Chem..

[23] Chetankumar K, Swamy BEK, Naik HSB (2021). MgO and MWCNTs amplified electrochemical sensor for guanine, adenine and epinephrine. Mater. Chem. Phys..

[24] Harisha KV, Swamy BEK, Ganesh PS, Jayadevappa H (2019). Electrochemical oxidation of haematoxylin at poly(alanine) modified carbon paste electrode: A cyclic voltammetric study. J. Electroanal. Chem..

[25] Chetankumar K, Swamy BEK (2019). Electrochemical investigation of catechol and hydroquinone at poly(o-phenylenediamine) modified carbon paste electrode: A voltammetric study. Anal. Bioanal. Electrochem..

[26] Teradale AB, Lamani SD, Ganesh PS, Swamy BEK, Das SN (2019). Poly-nile blue based electrochemical sensor for catechol and hydroquinone. Anal. Bioanal. Electrochem..

[27] Ganesh PS, Swamy BEK, Harisha KV (2017). Electropolymerisation of DL-methionine at carbon paste electrode and its application to the determination of catechol and hydroquinone. Anal. Bioanal. Electrochem..

[28] Chetankumar K, Swamy BEK, Sharma SC (2019). Poly (benzoguanamine) modified sensor for catechol in presence of hydroquinone: A voltammetric study. J. Electroanal. Chem..

[29] Kuskur CM, Swamy BEK, Jayadevappa H, Ganesh PS (2018). Ploy (rhodamine B) sensor for the norepinephrine and paracetamol: A voltammetric study. Ionics.

[30] Tekenya R, Pokpas K, Jahed N, Iwuoha EI (2019). Enhanced specificity and sensitivity for the determination of nickel(II) by square-wave adsorptive cathodic stripping voltammetry at disposable graphene-modified pencil graphite electrodes. Anal. Lett..

[31] Pandey A, Sharma S, Jain R, Raja AN (2020). Review-Pencil Graphite Electrode: An Emerging Sensing Material. J. Electroche. Soc..

[32] Chetankumar K, Swamy BEK, Sharma SC (2020). Fabrication of voltammetric efficient sensor for catechol, hydroquinone and resorcinol at MgO modified pre-treated carbon paste electrode. Mater. Chem. Phys..

[33] Mohanadas D, Tukimin N, Sulaiman Y (2019). Simultaneous electrochemical detection of hydroquinone and catechol using poly(3,4-ethylenedioxythiophene)/reduced graphene oxide/manganese dioxide. Synth. Met..

[34] Zhao L, Yu J, Yue S, Zhang L, Wang Z, Guo P, Liu Q (2018). Nickel oxide/carbon nanotube nanocomposites prepared by atomic layer deposition for electrochemical sensing of hydroquinone and catechol. J. Electroanal. Chem..

[35] Jiang D, Pang J, You Q, Liu T, Chu Z, Jin W (2019). Simultaneous biosensing of catechol and hydroquinone via a truncated cube-shaped Au/PBA nanocomposite. Biosens. Bioelectron..

[36] Dang Y, Wang X, Cui R, Chen S, Zhou Y (2019). A novel electrochemical sensor for the selective determination of hydroquinone and catechol using synergic effect of electropolymerized nicotinic acid film and Cd-doped ZnWO_4_ nanoneedle. J. Electroanal. Chem..

[37] Fan L, Li X, Kan X (2016). Disposable graphite paper based sensor for sensitive simultaneous determination of hydroquinone and catechol. Electrochim. Acta.

[38] Wang Z, Li M, Ye Y, Yang Y, Lu Y, Ma X, Zhang Z, Xiang S (2019). MOF-derived binary mixed carbon/metal oxide porous materials for constructing simultaneous determination of hydroquinone and catechol sensor. J. Solid State Electrochem..

[39] Charithra MM, Manjunatha JG (2019). Poly (l-Proline) modified carbon paste electrode as the voltammetric sensor for the detection of Estriol and its simultaneous determination with Folic and Ascorbic acid. Mater. Sci. Energy Technol..

[40] Fan J, Pang J, Zhang Y, Zhang L, Xua W, Wang J (2019). Simultaneous detection of hydroquinone and catechol with decreasing pH at a bare glassy carbon electrode surface. Anal. Methods.

[41] Zhang J, Song X, Ma S, Wang X, Wang W, Chen Z (2017). A novel sodium dodecyl benzene sulfonate modified expanded graphite paste electrode for sensitive and selective determination of dopamine in the presence of ascorbic acid and uric acid. J. Electroanal. Chem..

[42] Yin H, Zhang Q, Zhou Y, Ma Q, Liu T, Zhu L, Ai S (2011). Electrochemical behavior of catechol, resorcinol and hydroquinone at graphene–chitosan composite film modified glassy carbon electrode and their simultaneous determination in water samples. Electrochim. Acta.

[43] Chen Y, Liu XY, Zhang S, Yang LQ, Liu ML, Zhang YY, Yao SZ (2017). Ultrasensitive and simultaneous detection of hydroquinone, catechol and resorcinol based on the electrochemical co-reduction prepared Au-Pd nanoflower/ reduced graphene oxide nanocomposite. Electrochim. Acta.

[44] Buleandra M, Rabinca AA, Badea IA, Balan A, Stamatin I (2017). Voltammetric determination of dihydroxybenzene isomers using a disposable pencil graphite electrode modified with cobalt-phthalocyanine. Microchim. Acta.

[45] Daneshinejad H, Chamjangali MA, Goudarzi N, Amin AH (2018). Modification of glassy carbon electrode with poly(hydroxynaphthol blue)/multi-walled carbon nanotubes composite and construction a new voltammetric sensor for the simultaneous determination of hydroquinone, catechol, and resorcinol. Mater. Res. Express.

[46] Xu G, Tang B, Jing S, Tao J (2015). Simultaneous determination of hydroquinone, catechol and resorcinol at poly(3-thiophenemalonic acid) modified glassy carbon electrode. Int. J. Electrochem. Sci..

[47] Hong-Ying L, Lang-Lang Z, Zhi-Heng H, Yu-Bing Q, Han-Xiao X, Jia-Jun W, Wei-Wei X, Li-Hua L, Chun-Chuan G (2019). simultaneous detection of hydroquinone, catechol and resorcinol by an electrochemical sensor based on ammoniated-phosphate buffer solution activated glassy carbon electrode. Chin. J. Anal. Chem..

[48] Chetankumar K, Swamy BEK, Naik TSSK (2020). Electrochemical sensing of catechol in presence of hydroquinone using a carbon paste electrode amplified with poly (vanillin). Chem. Data Collect..

